# Supplementary material for: Yiai Fuzheng decoction inhibits triple-negative breast cancer by remodeling the immune microenvironment
Source: Front Immunol. 2025 Sep 30;16:1615631. doi: 10.3389/fimmu.2025.1615631 (PMC12518410; doi:10.3389/fimmu.2025.1615631)
Supplement: Supplementary file 2 [file DataSheet1.zip › Supplementary File 2/Treatment/C_vs_H/07_Pathway_Analysis/Pathway_Analysis/Sig_Pathways_Name/Butanoate metabolism.pdf]

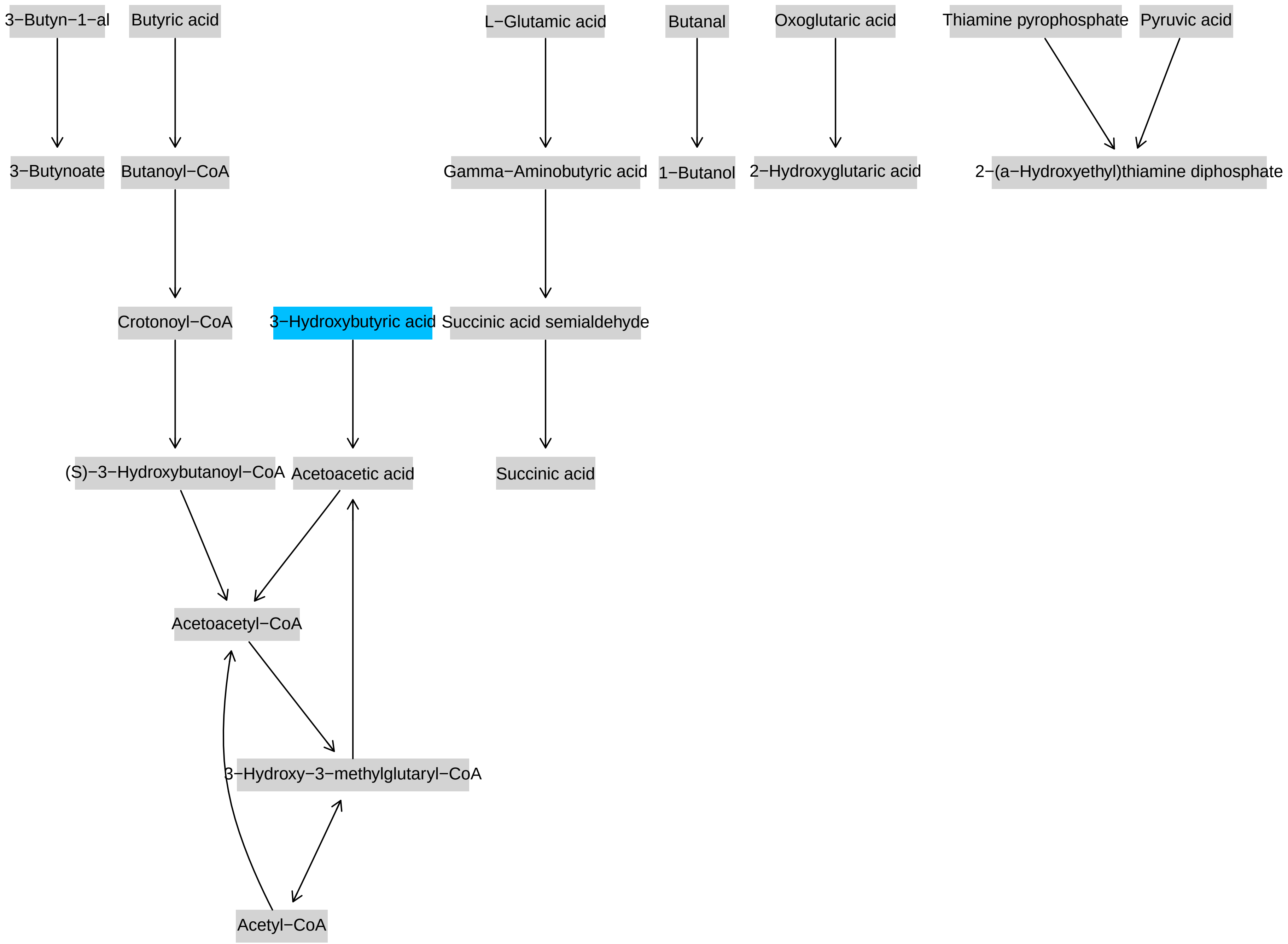
3−Butyn−1−al
Butyric acid
L−Glutamic acid
Butanal
Oxoglutaric acid
Thiamine pyrophosphate
Pyruvic acid
3−Butynoate
Butanoyl−CoA
Gamma−Aminobutyric acid
1−Butanol
2−Hydroxyglutaric acid
2−(a−Hydroxyethyl)thiamine diphosphate
Crotonoyl−CoA
3−Hydroxybutyric acid
Succinic acid semialdehyde
(S)−3−Hydroxybutanoyl−CoA
Acetoacetic acid
Succinic acid
Acetoacetyl−CoA
3−Hydroxy−3−methylglutaryl−CoA
Acetyl−CoA
